# Exploring the Role of Breast Density on Cancer Prognosis among Women Attending Population-Based Screening Programmes

**DOI:** 10.1155/2019/1781762

**Published:** 2019-11-27

**Authors:** Laia Domingo, Maria Sala, Javier Louro, Marisa Baré, Teresa Barata, Joana Ferrer, Maria Carmen Carmona-Garcia, Mercè Comas, Xavier Castells, CAMISS Study Group

**Affiliations:** ^1^Department of Epidemiology and Evaluation, IMIM (Hospital del Mar Medical Research Institute), Passeig Marítim, 25-29, 08003 Barcelona, Spain; ^2^Research Network on Health Services in Chronic Diseases (REDISSEC), Barcelona, Spain; ^3^Department of Paediatrics, Obstetrics and Gynaecology, Preventive Medicine and Public Health, Universitat Autònoma de Barcelona (UAB), 08193 Bellaterra, Barcelona, Spain; ^4^Cancer Screening and Clinical Epidemiology, Corporació Sanitària Parc Taulí, 08208 Sabadell, Spain; ^5^General Directorate of Health Care Programmes, Canary Islands Health Service, C/Juan XXIII, 13 35005, Las Palmas de Gran Canaria, Spain; ^6^Department of Radiology, Hospital de Santa Caterina, C/Dr. Castany, s/n, 17190 Salt, Girona, Spain; ^7^Epidemiology Unit and Girona Cancer Registry, Oncology Coordination Plan, Department of Health, Catalan Institute of Oncology, C/Sol, 15, Barcelona 17004, Girona, Spain; ^8^Girona Biomedical Research Institute (IDIBGI), C/Dr Castany s/n, 17190 Salt, Girona, Spain; ^9^Department of Medical Oncology, Catalan Institute of Oncology, University Hospital Dr. Josep Trueta, Av França s/n, 17007, Barcelona, Girona, Spain; ^10^IMIM (Hospital del Mar Medical Research Institute), Passeig Marítim, 25-29, 08003 Barcelona, Spain

## Abstract

**Background:**

Our aim was to assess the role of breast density on breast cancer mortality and recurrences, considering patient and tumour characteristics and the treatments received among women attending population-based screening programmes.

**Methods:**

We conducted a retrospective cohort study among women aged 50–69 years attending population-based screening programmes, diagnosed with invasive breast cancer between 2000 and 2009, and followed up to 2014. Breast density was categorised as low density (≤25% dense tissue), intermediate density (25–50%), and high density (≥50%). Cox proportional hazards regression models were fitted to estimate the adjusted hazard ratios (aHR) and 95% confidence intervals (95% CI) for death and recurrences, adjusting by patient characteristics, mode of detection (screen-detected vs. interval cancer), and tumour features.

**Results:**

The percentage of deaths and recurrences was higher among women with intermediate- and high-density breasts than among women with low-density breasts (*p*=0.011 for death; *p*=0.037 for recurrences). Adjusted Cox proportional hazards regression models revealed that women with intermediate- and high-density breasts had a higher risk of death than women with low-density breasts, being statistically significant for intermediate densities (aHR = 2.19 [95% CI: 1.16–4.13], aHR = 1.44 [95% CI: 0.67–3.1], respectively). No association was found between breast density and recurrences.

**Conclusions:**

Breast density was associated with a higher risk of death, but not of recurrences, among women participating in breast cancer screening. These findings reinforce the need to improve screening sensitivity among women with dense breasts and to routinely assess breast density, not only for its role as a risk factor for breast cancer but also for its potential influence on cancer prognosis.

## 1. Introduction

Mammographic breast density is defined as the relative amount of radiolucent elements (fatty tissue) and radiopaque elements of the breast (fibroglandular tissue). It has become a key element in breast cancer screening because of its dual effect on breast cancer risk: high breast density impairs the detection of abnormalities in the breast, decreasing the sensitivity of mammography [1], and is also an independent risk factor for breast cancer, as most cancers develop in the glandular parenchyma [2]. More recently, breast density has been postulated as a robust candidate for tailoring screening intervals, suggesting that annual screening may be more effective than biennial screening for women at high risk due to dense breasts in combination with other risk factors [3]. However, such an approach has not been implemented in any screening programme, since it requires more individual-level data, among many other unresolved issues and challenges [4].

Variations in breast density during a woman's lifetime may be influenced by several internal and external factors related to the hormonal environment. Breast density is inversely associated with age, with premenopausal women younger than 50 years being more likely to have dense breasts [5, 6]. The use of hormone replacement therapy slows the age-related trend to fatty tissue, especially for those women taking a combination of oestrogen and progestin components [7]. In addition, some studies have reported that tumours developing in dense breasts are more likely to express hormone receptors such as oestrogen receptor (ER) and progesterone receptor (PR) [8, 9], suggesting a positive association with stromal composition and the oestrogenic microenvironment.

However, whereas increased breast density is a well-recognised risk factor for breast cancer, the relationship between breast density and breast cancer prognosis is still controversial. Some studies have reported an increased risk of death for women with dense breasts [10, 11], while others have found an inverse association [12] or no relationship [13]. In addition, only few studies have been restricted to the context of mammography screening [14–16], also with contradictory results. Because this population has particular characteristics (e.g., average-risk women, women over 45/50 years, mostly postmenopausal), performing studies focused on this population may provide useful information to better understand the relationship between breast density and cancer prognosis and to eventually provide individually tailored screening strategies.

Our aim was to assess the role of breast density on mortality and recurrences, taking into account patient and tumour characteristics and the treatments received among women attending population-based screening programmes.

## 2. Materials and Methods

### 2.1. Setting and Study Population

This study was carried out among a retrospective cohort of 1,086 women with breast cancer, aged between 50 and 69 years, who underwent breast cancer screening in two Spanish regions (Catalonia and the Canary Islands; CAMISS retrospective cohort). All of them were diagnosed with breast cancer between 2000 and 2009 and were followed up until June 2014. The study included asymptomatic women with cancers detected in routine screening mammograms and symptomatic women with cancers detected between two screening mammograms (interval cancers).

Mammography screening in Spain follows the recommendations of the European Guidelines for quality assurance in breast cancer screening and diagnosis [17], offering all women aged 50 to 69 years free biennial screening. Two mammographic projections (mediolateral oblique and craniocaudal views) are made, using the BI-RADS (Breast Imaging Reporting and Data System) classification for mammogram reading [18].

As breast density is not routinely recorded by all participating screening programmes, we determined breast density for a subsample of cases. Sample size was calculated to estimate a hazard ratio of 2.5 [15], with a mortality rate of 14.5% (from the whole CAMISS cohort). With 5% significance level and 80% power, 55 subjects were needed in the high-density group. The subsample included all interval cancers with available screening and diagnostic mammograms and a random sample of screen-detected cancers, matched by screening programme and year of cancer diagnosis. After the breast density assessment, this resulted in 375 invasive breast cancers, 79 of them assigned to the high-density group, thus assuring enough sample size for the analysis.

The study protocol was approved by the Ethics Committee of Parc de Salut Mar, Barcelona (CEIC Parc de Salut Mar). Specific patient consent was not required.

### 2.2. Breast Density Assessment

For the purpose of this study, breast density was retrospectively evaluated by three experienced radiologists who followed a consensus-based protocol, as detailed elsewhere [19]. In brief, each radiologist determined the breast density of the cancer-free breast at the moment of diagnosis using Boyd's scale, a semiquantitative score of six categories using percentages of density: A: 0%; B: 1–10%; C: 10–25%; D: 25–50%; E: 50–75%; F: 75–100% [20]. For statistical purposes, breast density was collapsed into low (≤25% density), intermediate (25–50% density), and high density (≥50% density).

### 2.3. Study Variables

Patient information, including age at diagnosis, menopausal status, hormone replacement therapy (ever/never), and first-degree family history of breast cancer, was obtained from the databases of the screening programmes. To obtain information on the burden of disease at diagnosis, we manually reviewed clinical records to identify the presence of comorbidities and construct the Charlson comorbidity index (CCI) [21]. The CCI was stratified into three categories: CCI = 0, CCI = 1, and CCI ≥ 2.

Information on mode of detection was obtained from the screening programme databases and by merging data with population-based cancer registries, the hospital minimum basic dataset, and hospital-based cancer registries. We differentiated between breast cancers detected by routine screening mammograms (i.e., screen-detected cancers) and cancers detected between 2 screening mammograms, or within 24 months for women who reached the upper age limit (i.e., interval cancers). Further details on the identification of interval cancers are explained elsewhere [19]. Tumour-related information, including tumour size, lymph node involvement, focality, histological type, histological grade, and biomarker expression, was retrieved from the cancer registries, hospital-based registries, and clinical records. Biomarker expression included information on ER, PR, human epidermal growth factor receptor 2 (Her2), p53, and Ki67 status. The positivity criteria for biomarker expression followed international recommendations and their updates throughout the study period [22, 23]. Tumours were classified into the following four phenotypes based on the expression of ER, PR, and Her2: (1) luminal A: ER+/Her2− or PR+/Her2−; (2) luminal B: ER+/Her2+ or PR+/Her2+; (3) Her2: ER−/PR−/Her2+; and (4) triple-negative: ER−, PR−, Her2− [24].

From the review of the clinical records, we obtained information on the treatments received. We considered two types of surgery: radical (including all the mastectomies performed, whether radical or simple) and conservative. Information on breast surgery and axillary lymph node dissection (ALND) treatments was collapsed into a single explicative variable. Information on adjuvant treatment was categorised as follows: chemotherapy, radiotherapy, and hormonal therapy; radiotherapy and hormonal therapy; and other treatments.

### 2.4. Follow-Up Information

Information on recurrences (including locoregional and distant recurrences), second breast neoplasms, and vital status at the end of follow-up (alive or dead) was obtained from the cancer registries and clinical records. Locoregional recurrence was defined as disease recurrence within the ipsilateral breast or chest wall, in the ipsilateral axillary nodes, internal mammary nodes, or supraclavicular nodes. Distant recurrence was defined as disease recurrence in sites other than the breast or regional lymph nodes (bone, skin, or visceral metastasis). A second neoplasm was considered as a second primary carcinoma developing in the ipsilateral or contralateral breast.

Overall survival was computed from the date of breast cancer diagnosis to death from any cause. Patients were censored at the date of their last hospital visit. Recurrence-free survival was computed from the date of breast cancer diagnosis to the first locoregional or distant recurrence, whichever occurred first. Women lost to follow-up or those who died were censored either at the last visit or at death. The median follow-up period was 8.7 years (interquartile range (IQR): 7.2–10.6).

### 2.5. Statistical Analyses

Descriptive analyses of patient and tumour characteristics and the treatments received according to breast density categories were explored using contingency tables.

Survival curves for overall mortality and for recurrences were generated by using the Kaplan–Meier method and were compared by the log-rank test. Recurrence-free survival and overall survival were plotted by breast density categories. 5-year and 10-year survival rates and their 95% confidence intervals (95% CI) were computed.

We fitted two multivariate Cox proportional hazards regression models to estimate the hazard ratios (HR) and their 95% CI for death and recurrences using a stepwise backward variable selection approach. The initial model included all predictors. In the final models, we forced to include age, screening programme, and CCI as adjusting variables, although they were not statistically significant. The proportional hazards assumption was ascertained by assessment of log-log survival plots. To test the statistical significance of breast density variable as a whole, we performed a Wald test in both models.

All statistical tests were two-sided. *p* values <0.05 were considered statistically significant. Analyses were performed using the statistical software IBM SPSS Statistics version 23.0 (Armonk, NY, USA) and R statistical software version 3.3.2 (http://www.r-project.org).

## 3. Results

A total of 375 invasive breast cancers were included in this study, most of them detected among women with low-density breasts (51.2%, 27.7%, and 21.1% of tumours detected in women with low-, intermediate-, and high-density breasts).

Patient characteristics by breast density categories are summarized in Table 1. Percentages of women with low breast density were highest among older and postmenopausal women. No differences were observed between a family history of breast cancer, the use of hormone replacement therapy or comorbidities, and breast density categories.

Tumour characteristics according to breast density categories are shown in Table 2. Screen-detected cancers were more common among women with low-density breasts, whereas interval cancers were more frequent in intermediate- and high-density breasts. Tumours detected in low-density breasts showed a trend to be smaller, node-negative, unifocal, and triple-negative. No differences were observed among the treatments received, although the percentage of radical surgery tended to be higher among women with dense breasts.

**Figure 1:**
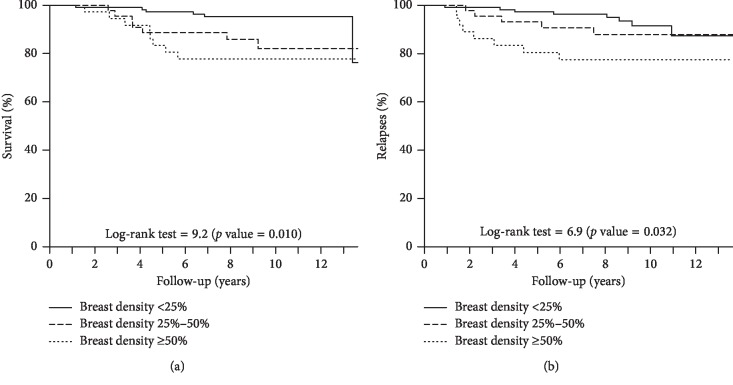
Survival and recurrence-free survival by breast density. (a) Overall survival; log-rank test = 0.010. (b) Recurrence-free survival; log-rank test = 0.032.

Kaplan–Meier curves revealed poorer overall survival (*p*=0.010) and poorer relapse-free survival (*p*=0.032) among women with high-density breasts (Figure 1). 5-year overall survival rate for women with low breast density was 0.97 (95% CI: 0.94–1.00), whereas figures for women with high breast density were 0.83 (95% CI: 0.72–0.96). The same pattern was observed at 10 years of follow-up. Recurrence-free survival rate at 5 years was 0.97 (0.94–1.00) for women with low breast density and 0.81 (0.69–0.95) for women with high breast density (Table 3).

Adjusted Cox proportional hazards regression models revealed that breast density was statistically significant for predicting mortality (Wald test *p* value = 0.050) but not for predicting recurrences (Wald test *p* value = 0.499). Women with intermediate- and high-density breasts had a higher risk of death than women with low-density breasts, reaching statistical significance for intermediate densities (Table 4) (aHR = 2.19 [95% CI: 1.16–4.13], aHR = 1.44 [95% CI: 0.67–3.10], for intermediate and high densities, respectively). Tumours arising as interval cancers (aHR = 1.96 [95% CI: 1.09–3.52] and node-positive tumours were also associated with a higher risk of death (aHR = 2.73 [95% CI: 1.55–4.81]) in the adjusted model (data not shown).

Breast density showed no association with the risk of recurrences (aHR = 1.43 [95% CI: 0.71–2.89]; aHR = 1.47 [95% CI: 0.71–3.08], for intermediate and high densities, respectively) (Table 5). Node-positive tumours showed an increased risk of recurrences in the adjusted analysis (aHR = 3.96 [95% CI: 2.12–7.39]) (data not shown).

## 4. Discussion

The results of the current study suggest that higher breast density is associated with a greater risk of death in women participating in breast cancer screening, while breast density showed no association with the risk of recurrences.

The positive association between dense tissue and risk of death is consistent with some [14, 15], but not all [16, 25], prior studies conducted among screened women. Based on the data of Swedish women, Chiu et al. found that dense tissue increased mortality from breast cancer in addition to increasing breast cancer risk and the likelihood of more aggressive tumours [14]. Based on Danish data, Olsen et al. also found a positive association between dense tissue and death, although they reported lower case fatality among tumours developing in dense breasts [15]. By contrast, a study carried out in the UK [25] reported no relationship between breast density and survival. In that study, however, the screening interval was 3 years, and the survival analyses were not adjusted. A recent study carried out among the Dutch population also reported no relationship between breast density and survival [16]. In this study, as pointed out by the authors, the lack of tumour-related information may confound the results shown. In addition, the definition for high density includes ≥25% of dense tissue, differing from most of the published studies. Other works conducted in nonscreening populations have also found contradictory results, some of them reporting positive associations between breast density and mortality [10, 11] and others finding no association [13] or even a negative association [12].

Some authors have hypothesized that the association between higher density and worse survival would be explained by the diagnosis delay due to the masking effect. In that sense, we do observe a higher percentage of larger, node-positive, and interval cancers among women with high-density breasts in the descriptive data. In the adjusted analyses, lymph node involvement and detection as an interval cancer were also associated with mortality, along with intermediate breast densities so that the current results would support this hypothesis, since breast density as well as other factors related to diagnostic delay remained associated with the risk of death. These findings reinforce the need to improve screening sensitivity among women with dense breasts, which is currently been proposed by means of shifting the conventional one-size-fits-all screening approach towards more personalized screening strategies based on the individual risk of breast cancer.

Other authors have postulated that the relationship between breast density and survival may be explained by the tumour characteristics of cancers arising in epithelial tissue. It has been suggested an increased proliferation and growth factors in dense tissue [26, 27] that may be involved in pathways that lead to more aggressive tumours. Nevertheless, the evidence supporting this hypothesis is not conclusive and seems contradictory to the overrepresentation (although nonsignificant) of triple-negative cancers in low-density breasts, observed in the current descriptive data and in previous works [19, 28]. Further studies conducted in larger cohorts, with information on breast density, tumour characteristics, and clinical outcomes, are warranted to elucidate the mechanisms through which breast density and prognosis are associated.

Contrasting with prior series, we did not find association between breast density and the risk of recurrences [29–31]. Those studies reported an increased risk of locoregional recurrences, but not for distant metastasis or death. Unfortunately, our study sample was not large enough to replicate the analysis for different types of recurrence. Besides, the populations considered in these works differed from ours, since they included study periods prior to ours, which could involve different treatment schemes. In addition, the study by Park et al. [30] only included patients undergoing breast-conserving surgery and radiotherapy, whereas we included both patients receiving and not receiving radiotherapy, which is strongly related to the risk of recurrences [29]. Our adjusted analyses revealed that the only factor associated with recurrences was the lymph node involvement at diagnosis.

Our study is limited by the relatively small number of events in some categories, which prevented us from including all breast density categories of Boyd's scale in the adjusted model. Nevertheless, most studies assessing the effect of breast density on mortality outcomes collapse breast density data into two or three categories, making our data more comparable with those of previous works. Second, we were not able to explore breast-specific cancer mortality. Previous studies exploring both breast-specific cancer mortality and mortality from other causes found that the latter was not associated with breast density [11]. Therefore, the impact of analysing all causes of death together may lead to underestimation of the effect of breast density on mortality. Finally, we used a qualitative classification for breast density assessment, which is known to have moderate interobserver concordance [32]. However, to minimise misclassification, breast density assessment was centralised and performed by a panel of experienced radiologists, specially trained for the study [19].

The current study is strengthened by the homogeneity of the study population included. Restricting the study to screening participants allowed us to explore the effect of breast density on a relatively homogeneous group of patients in terms of age range and tumour stage. Thus, the conclusions drawn from the current work are robust and informative within the framework of population-based screening and are of interest for tailored screening strategies. In addition, the availability of data on comorbidities, patient and tumour characteristics, and the treatments received allowed us to control for important prognostic factors and to explore—for the first time among women participating in breast cancer screening—the effect of breast density on mortality considering both patient and tumour characteristics and treatments received.

## 5. Conclusion

In conclusion, our findings reveal that increased breast density was associated with worse survival outcomes among women participating in breast cancer screening. This association seems to be mainly explained as a result of the masking effect of dense tissue, although an underlying biological mechanism in the stroma composition may also play a role. These findings reinforce the need to improve screening sensitivity among women with dense breasts by means of more personalized screening approaches as well as the importance to routinely assess and record information on breast density during the screening process, both because of its utility as a predictive factor for breast cancer and because of its role in breast cancer prognosis.

## Figures and Tables

**Table 1:** Patient-related characteristics by breast density categories.

	Total *n* = 375 (%)	Low breast density (<25%) *n* = 192 (%)	Intermediate breast density (25–50%) *n* = 104 (%)	High breast density (>50%) *n* = 79 (%)
Age groups (years)				
50–54	106 (28.3)	34 (17.7)	33 (31.7)^a^	39 (49.4)
55–59	102 (27.2)	50 (26)	33 (31.7)	19 (24.1)
60–64	103 (27.5)	64 (33.3)	24 (23.1)	15 (19)
65–70	64 (17.1)	44 (22.9)	14 (13.5)	6 (7.6)
Menopausal status				
Premenopause	31 (13.3)	5 (4.7)	9 (12.3)	17 (32.1)
Menopause	202 (86.7)	102 (95.3)	64 (87.7)	36 (67.9)
Hormone replacement therapy				
No	192 (85)	86 (84.3)	60 (84.5)	46 (86.8)
Yes	34 (15)	16 (15.7)	11 (15.5)	7 (13.2)
Family history of breast cancer				
No	200 (86.6)	90 (85.7)	63 (86.3)	47 (88.7)
Yes	31 (13.4)	15 (14.3)	10 (13.7)	6 (11.3)
Charlson comorbidity index				
0	285 (76)	143 (74.5)	79 (76)	63 (79.7)
1	53 (14.1)	31 (16.1)	13 (12.5)	9 (11.4)
2	37 (9.9)	18 (9.4)	12 (11.5)	7 (8.9)

**Table 2:** Tumour characteristics by breast density categories.

	Total *n* = 375 (%)	Low breast density (<25%) *n* = 192 (%)	Intermediate breast density (25–50%) *n* = 104 (%)	High breast density (>50%) *n* = 79 (%)
Mode of detection				
Screen-detected cancers	195 (52)	113 (58.9)	45 (43.3)	37 (46.8)
Interval cancer	180 (48)	79 (41.1)	59 (56.7)	42 (53.2)
Tumour size				
<20 mm	199 (61.8)	108 (56.3)	53 (51)	38 (48.1)
≥20 mm	123 (38.2)	58 (30.2)	33 (31.7)	32 (40.5)
Lymph node involvement				
Negative	218 (66.1)	117 (60.9)	60 (57.7)	41 (51.9)
Positive	112 (33.9)	51 (26.6)	30 (28.8)	31 (39.2)
Focality				
Unifocal	301 (84.6)	155 (87.1)	80 (80)	66 (84.6)
Multifocal and/or multicentric	55 (15.4)	23 (12.9)	20 (20)	12 (15.4)
Histological type				
Ductal	303 (80.8)	152 (79.2)	86 (82.7)	65 (82.3)
Lobular	39 (10.4)	22 (11.5)	8 (7.7)	9 (11.4)
Others	33 (8.8)	18 (9.4)	10 (9.6)	5 (6.3)
Histological grade				
I	83 (23.9)	44 (22.9)	18 (17.3)	21 (26.6)
II	134 (38.5)	64 (33.3)	40 (38.5)	30 (38)
III	116 (33.3)	60 (31.3)	35 (33.7)	21 (26.6)
NA	15 (4.3)	9 (4.7)	3 (2.9)	3 (3.8)
Oestrogen receptor				
Negative	88 (23.5)	46 (24)	28 (26.9)	14 (17.7)
Positive	287 (76.5)	146 (76)	76 (73.1)	65 (82.3)
Progesterone receptor				
Negative	150 (40.1)	70 (36.5)	45 (43.3)	35 (44.3)
Positive	224 (59.9)	121 (63)	59 (56.7)	44 (55.7)
HER2				
Negative	261 (79.1)	133 (69.3)	70 (67.3)	58 (73.4)
Positive	69 (20.9)	35 (18.2)	21 (20.2)	13 (16.5)
Ki67				
Negative	114 (60.3)	62 (32.3)	29 (27.9)	23 (29.1)
Positive	75 (39.7)	49 (25.5)	15 (14.4)	11 (13.9)
Tumour phenotype				
Luminal A	155 (48)	79 (41.1)	38 (36.5)	38 (48.1)
Luminal B	93 (28.8)	46 (24)	26 (25)	21 (26.6)
HER2	31 (9.6)	11 (5.7)	12 (11.5)	8 (10.1)
Triple-negative	44 (13.6)	29 (15.1)	11 (10.6)	4 (5.1)
Treatment				
Conservative surgery only or with sentinel lymph node biopsy	97 (26.4)	50 (26.4)	28 (28)	19 (24.1)
Conservative surgery with axillary lymph node dissection	182 (49.5)	100 (52.9)	44 (44)	38 (48.1)
Radical surgery with or without lymphadenectomy	79 (21.5)	35 (18.5)	24 (24)	20 (25.3)
No surgery and/or adjuvant treatment	10 (2.7)	4 (2.1)	4 (4)	2 (2.5)
Adjuvant treatment after surgery				
Chemotherapy, radiotherapy, and hormonal therapy	122 (32.5)	54 (28.1)	36 (34.6)	32 (40.5)
Radiotherapy and hormonal therapy	123 (32.8)	68 (35.4)	35 (33.7)	20 (25.3)
Other treatments	130 (34.7)	70 (36.5)	33 (31.7)	27 (34.2)

**Table 3:** 5-year and 10-year survival rates for overall survival and recurrence-free survival.

	5-year survival rate (95% CI)	10-year survival rate (95% CI)
Overall survival		
Low breast density (<25%)	0.97 (0.94–1.00)	0.95 (0.91–0.99)
Intermediate breast density (25–50%)	0.89 (0.80–0.99)	0.82 (0.71–0.96)
High breast density (>50%)	0.83 (0.72–0.96)	0.78 (0.65–0.93)
Recurrence-free survival		
Low breast density (<25%)	0.97 (0.94–1.00)	0.92 (0.85–0.98)
Intermediate breast density (25–50%)	0.93 (0.86–1.00)	0.88 (0.79–0.99)
High breast density (>50%)	0.81 (0.69–0.95)	0.78 (0.65–0.93)

**Table 4:** Unadjusted and adjusted hazard ratios for death.

	Number of deaths	Unadjusted HR	Adjusted HR^*∗*^
Breast density			
<25%	20	Ref.	Ref.
25–50%	24	2.48 (1.37–4.49)	2.19 (1.16–4.13)
>50%	15	1.89 (0.97–3.7)	1.44 (0.67–3.10)

^*∗*^The final model included breast density, mode of detection, lymph node involvement, age, Charlson comorbidity index, and screening programme. HR: hazard ratio; aHR: adjusted hazard ratio.

**Table 5:** Unadjusted and adjusted hazard ratios for recurrences.

	Number of recurrences	Unadjusted HR	Adjusted HR^*∗*^
Breast density			
≤25%	19	Ref.	Ref.
25–50%	16	1.72 (0.89–3.35)	1.43 (0.71–2.89)
≥50%	17	2.34 (1.21–4.5)	1.47 (0.71–3.08)

^*∗*^Adjusted by breast density, progesterone receptor, lymph node involvement, age, Charlson comorbidity index, and screening programme. HR: hazard ratio; aHR: adjusted hazard ratio.

## Data Availability

The retrospective observational data used to support the findings of this study are available from the corresponding author on reasonable request.

## Abbreviations

ALND: Axillary lymph node dissection
BI-RADS: Breast Imaging Reporting and Data System
CCI: Charlson comorbidity index
ER: Oestrogen receptor
Her2: Human epidermal growth factor receptor 2
HR: Hazard ratios
IQR: Interquartile range
PR: Progesterone receptor
TNM: Tumour-node-metastasis.
